# Pediatric Germ Cell Tumors: A Developmental Perspective

**DOI:** 10.1155/2018/9059382

**Published:** 2018-02-04

**Authors:** Joshua L. Pierce, A. Lindsay Frazier, James F. Amatruda

**Affiliations:** ^1^Department of Pediatrics, University of Texas Southwestern Medical Center, Dallas, TX 75390, USA; ^2^Department of Molecular Biology, University of Texas Southwestern Medical Center, Dallas, TX 75390, USA; ^3^Department of Pediatric Oncology, Children's Hospital Dana-Farber Cancer Care, Boston, MA 02115, USA; ^4^Department of Internal Medicine, University of Texas Southwestern Medical Center, Dallas, TX 75390, USA

## Abstract

Germ cell tumors (GCTs) arising in infants, children, and adolescents present a set of special challenges. GCTs make up about 3% of malignancies in children aged 0–18 and nearly 15% of cancers in adolescents. Epidemiologic and molecular evidence suggests that GCTs in young children likely represent a distinct biologic group as compared to GCTs of older adolescents and adults. Despite this difference, pediatric GCTs are typically treated with cisplatin-based multiagent regimens similar to those used in adults. There is evidence that children are particularly vulnerable to late effects of conventional therapy, including ototoxicity, pulmonary abnormalities, and secondary malignancies, motivating the search for molecular targets for novel therapies. Evidence is accumulating that the genes and mechanisms controlling normal germ cell development are particularly relevant to the understanding of germ cell tumorigenesis. Perturbations in the epigenetic program of germ cell differentiation, with resulting effects on the regulation of pluripotency, may contribute to the marked histologic variability of GCTs. Perturbations in the KIT receptor signaling pathway have been identified via next-generation sequencing studies and in genome-wide association studies of testicular cancer susceptibility. Here, we review these and other biological insights that may fuel further translational and clinical research in childhood GCTs.

## 1. Introduction

“Pediatric germ cell tumor” is the term used to describe malignant cancers of germline cells in patients aged 0–18 years. These cancers may arise in the testis, the ovary, or the extragonadal sites including the sacrococcygeal area and the mediastinum. Germ cell tumors (GCTs) also occur in the brain in children and young adults. Though intracranial GCTs (iGCTs) are histologically similar to extracranial GCTs, it is unclear if tumors in the different sites arise by similar or different mechanisms, and the treatment approaches used are somewhat different; for these reasons, iGCTs are not further considered here.

Though the biology and clinical presentation of pediatric GCTs share significant overlap with that of adult testicular (T) GCTs, there are important differences that should be kept in mind. First, epidemiologic data reveal two distinct peaks in GCT incidence, one in young children (aged approximately 0–4 years) and a second peak beginning in puberty [1]. While the histologic presentation and molecular biology of GCTs arising in adolescents appear similar to those in adult TGCTs, germ cell tumors in very young children have important differences (reviewed below), suggesting that they may represent a distinct disease. Altogether GCTs make up about 3% of malignancies in children aged 0–18 and incidence rises with onset of puberty, GCTs account for 15% of the malignancies diagnosed during adolescence. As with adult TGCTs, the mainstay of treatment for pediatric GCTs is cisplatin-based multiagent regimens that have proved to be highly effective even in the setting of advanced disease. However, increasing evidence has emerged of adverse late effects in adult male survivors of TGCT, including a doubling of the risk of early-onset cardiovascular disease [2] and second malignancies [3, 4]. It is worth noting that GCTs were not included in the malignancies studied in the landmark Children's Cancer Survival Study, and thus, the potential long-term toxicities of conventional chemotherapy in pediatric GCT patients are still largely unknown. There is evidence that children are particularly vulnerable to late effects of therapy, especially ototoxicity and pulmonary abnormalities [5]. In a large cohort study, the cumulative risk of secondary malignancy also increased with decreasing age at diagnosis [4].

Finally, treatment regimens for pediatric GCT have largely been based on clinical trial results from adult men with TGCT, who represent the largest patient population. Whether these results apply equally to children with gonadal or extragonadal GCT remains to be established. In recent years, investigators in the Children's Oncology Group (USA) and the Children's Cancer and Leukaemia Group (UK) have joined forces to form the Malignant Germ Cell Tumor International Collaborative (MaGIC) Consortium to improve outcomes for patients with germ cell tumors (GCTs) by generating new insights into etiology, prognosis, toxicity reduction, and optimal treatment. MaGIC investigators have produced a revised evidence-based risk stratification for pediatric and adolescent GCTs based on amalgamation of 25 years of clinical trial data from the US and the UK [6] that separated patients into low-, standard-, and poor-risk groups. This risk stratification has in turn informed the development of the clinical trial AGCT1531 recently opened by the Children's Oncology Group (https://clinicaltrials.gov/show/NCT03067181), which aims to eliminate unnecessary chemotherapy in patients with Stage I disease at all sites (testicular, ovarian, and extragonadal) likely cured with surgery alone who will undergo active surveillance. AGCT1531 will also test whether the less-toxic carboplatin can be substituted for cisplatin in standard-risk patients. The COG will also be opening in the near future a trial for poor-risk patients, testing the efficacy of standard BEP chemotherapy versus accelerated BEP given every 2 weeks instead of every 3 weeks. This trial is conducted by the Australian and New Zealand Urogenital and Prostate Cancer Trials Group (ANZUP). The clinical management of pediatric GCTs was recently comprehensively reviewed [7].

## 2. Histologic Presentation of Germ Cell Tumors

**Figure 1:**
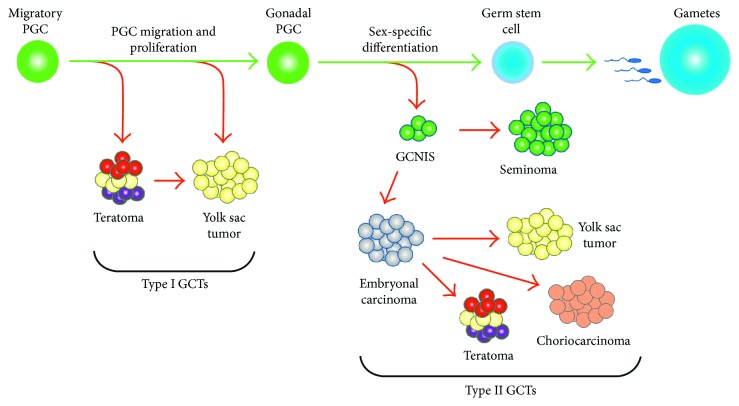
Germline development and histologic subtypes of GCTs. Primordial germ cells (PGCs) are specified early in embryogenesis and migrate through the embryo to the developing gonad. Type I GCTs exhibit a limited histologic spectrum, a partial erasure of genomic imprinting, and a propensity for development at extragonadal sites, all suggesting a derivation from early stages of germ cell development. Type II GCTs frequently contain foci of germ cell neoplasia in situ (GCNIS) and exhibit the full range of seminoma and nonseminoma histologies. Together with a more complete erasure of imprinting, these features suggest that the Type II tumors arise at a later stage of germline development.

Several lines of evidence suggest that GCTs do not arise from a mature gonadal cell (e.g., a spermatogonial stem cell) but rather from a germ cell in early stages of development. This was discovered through an interesting set of observations that linked etiological phenomena to characteristics of the developing germline [8]. GCTs can be classified into two major types based on histology, known as seminomatous GCTs and nonseminomatous GCTs (Figure 1). Seminomatous GCTs are tumors which are made of undifferentiated germ cells which can histologically resemble early spermatogonia, oogonia, or even germ cells from developmental lineages. These tumors are called seminoma when present in the testis, dysgerminoma when present in the ovary, and germinoma when found in an extragonadal site. Nonseminomatous GCTs can be further subdivided into the distinct histologies of embryonal carcinoma (EC), yolk sac tumor (YST), teratoma (TER), and choriocarcinoma (CC). EC comprised undifferentiated cells that histologically resemble embryonic cells from the blastocyst. YST is the most common malignant GCT of young children. These tumors have a very complex endodermal morphology with components of both embryonic and extraembryonic endoderm. TER histologically presents as a disordered mixture of differentiated cell types from all three somatic germ layers. In some cases, a component of a TER may acquire a unique neural differentiation state, known as an immature teratoma. CC is a rare histology and represents trophoblastic differentiation. In general, NSGCTs are more likely to be resistant to standard therapies. GCTs may present as a pure form with only one histology or as an amalgam of multiple types, known as mixed malignant germ cell tumor (MMGCT).

GCTs arising in children and adolescents can be further classified into two types depending on the age at presentation and histologic features [9]. Type I tumors generally present in children less than 4 years of age and may present as TER, YST, or mixtures of the two. Type II tumors arise around the time of puberty up through young adulthood and exhibit the full range of seminomatous and nonseminomatous histologies. Type II GCTs are often associated with a presumptive precursor lesion known as germ cell neoplasia in situ (GCNIS). GCNIS is a histologically diagnosed cell pattern usually seen in the normal tissue adjacent to the tumor [10] and is composed of undifferentiated germ cells that have proliferated within a seminiferous tubule [11]. GCNIS is not observed adjacent to Type I tumors, which are thus designated as non GCNIS-associated GCTs. These differences, along with molecular features discussed below, suggest that Type I and Type II GCTs may develop from germ cells at different stages of development.

## 3. Molecular Genetics of Pediatric GCTs

The most frequent chromosomal aberrations in Type II GCTs are amplification of chromosome 12p, usually through creation of isochromosome 12p, and amplification of the X chromosome. However, X amplification is an uncommon event in pediatric germ cell tumors, and 12p gain, while present, is infrequent [12]. Instead, pure YST, the most common malignant Type I GCT, has been shown to most commonly possess gain of 1q, 11q, 20q, and 22 and loss of 1p, 6q, and 16q [13]. Germ cell tumors also exhibit loss of genomic imprinting, which is partial in Type I GCTs and more complete in Type II GCTs. Since primordial germ cells undergo erasure of imprinting during germline development, this difference further suggests that Type I GCTs may arise from an earlier stage of development compared to Type II GCTs [14].

While the spectrum of somatic mutations in TGCTs is beginning to be defined (reviewed in this issue by Woldu et al.), little is known about the mutational status of pediatric GCTs. Addeo et al. found that *BAX* mutations in pediatric GCTs correlated with outcome [15]. At the transcriptional level, Palmer and coworkers found that Type I and Type II GCTs exhibit distinct gene expression profiles, even when controlling for histologic subtype [16]. Another important finding within GCTs is that the landscape of microRNAs (miRNAs) is changed relative to the normal gonad [17]. Palmer and coworkers demonstrated that the eight main miRNAs from the miR-371–373 and miR-302/367 clusters were overexpressed in all malignant GCT tissues [18]. Murray et al. further showed that levels of miRNAs from these two clusters were elevated in the serum at the time of diagnosis of an extragonadal malignant GCT, with levels falling and remaining low during uneventful clinical follow-up [19]. These serum findings were confirmed in extracranial malignant GCTs across a range of representative ages (pediatric/adult), anatomical sites, and histological subtypes, the majority of which were tumor marker negative [17]. These and subsequent studies suggest a possible role for miRNA profiling in GCT diagnosis and risk stratification.

## 4. Pediatric Germ Cell Tumors as a Developmental Disease: Vulnerabilities in Primordial Germ Cells

The primordial germ cell (PGC), responsible for specification of the germline, is unique among cells of the body in its requirement to maintain the pluripotent potential necessary for gamete generation. This requirement creates a unique developmental cycle which involves stages of vulnerability to improper differentiation. The PGC must be specified from the rest of the developing embryo through genetic and epigenetic events; it subsequently migrates throughout the body to the site of the gonad, and it must then undergo sex specific differentiation. Each of these stages reflects both a developmental feature and a clue related to the phenotypes and characteristics of germ cell tumors.

## 5. PGC Specification and Pluripotency

The first event in the development of the germline is the specification of PGCs in the early embryo. This specification event endows the PGCs with pluripotency as marked by a unique histological and genetic signature. PGC specification in humans occurs at ∼2 weeks after fertilization when BMP signals target to the mesendoderm of the preprimitive streak embryo, which leads to the induction of SOX17 which, with BLIMP1, suppresses endodermal differentiation and activates PGC differentiation [20]. These cells express the pluripotency-associated markers OCT3/4, LIN28A, and NANOG histologically and begin a process of global DNA demethylation necessary for the later establishment of sex-specific gametic imprinting [21–23]. This genetic and epigenetic state creates the platform for development of the gamete, which leads to formation of the totipotent zygote after fertilization.

This unique developmental licensing of pluripotency is likely partially responsible for the extremely heterogeneous histological variation amongst germ cell tumors. Unlike most other tumor types, germ cell tumors may present with cells from each of the three germ layers as well as undifferentiated germline and extraembryonic cells [24]. In addition to GCTs exhibiting histological heterogeneity, it has been shown that both seminomas and nonseminomas retain pluripotency-associated protein expression (SALL4, OCT3/4, and LIN28A), which indicates the pluripotent growth potential even amongst the different differentiation states [24]. Furthermore, it has been shown that LIN28A expression in malignant GCTs is responsible for maintaining an undifferentiated state through repression of the tumor suppressive let-7 miRNA family [25]. Additionally, the protein EPCAM, which is expressed in undifferentiated embryonic stem cells (ESCs), is associated with malignant nonseminomatous GCTs and has been suggested as a serum diagnostic marker in the treatment of GCTs [26, 27].

The second step of PGC specification, global demethylation, is another hallmark of GCTs and may be related to GCT susceptibility [20]. GWAS studies have revealed a GCT-associated SNP near the gene *PRDM14*, a key PGC pluripotency marker in mice [28, 29]. Though the function of PRDM14 is still being elucidated in humans, it is possible that it is involved in maintenance of the pluripotent state through the initiation of global demethylation, as it is in mice [30]. DNA demethylation at this stage allows for biallelic expression of genes [31]. Research on methylation states of the gene *SNRPN* revealed that most GCTs possess hypomethylation which lends credence to the idea that GCTs were derived from PGCs [32]. In addition to connecting GCTs to PGCs, this loss of transcriptional control likely provides a vulnerability in GCT development as loss of imprinting is a common event in many cancers [33]. This open epigenetic environment is thought to be protected by activation of the repressive chromatin modifications H3K27me3 and H3K9me3 concurrent with global demethylation. However, another testis cancer GWAS SNP near *ATF7IP*, a gene related to chromatin dynamics, may play a role in disrupting this repressive mark [34, 35]. Disregulation of ATF7IP with its cofactor SETDB1 may interfere with proper repressive chromatin marks, as it is known to be involved in creation of H3K9me3 marks, though further work is needed to establish this connection [36, 37]. It is likely that the pluripotent specification of PGCs as well as the physiological loss of imprinting naturally provides two pro-oncogenic conditions that may be co-opted by improper differentiation cues.

This creation and maintenance of the pluripotent state prior to and during migration may explain the histologies specific to Type I GCTs, namely teratoma (TER) and yolk sac tumor (YST) [24]. As mentioned previously, TER frequently presents in a mature form with fully differentiated cell types. It is likely that these tumors are the result of improper regulation of pluripotency in which reacquisition of epiblast-like pluripotency during improper PGC specification results in cells that attempt to recapitulate embryonic development, including creation of the three germ layers. Cells in teratomas may not, however, receive the very specific spatial and temporal differentiation cues needed for proper embryogenesis, resulting in disorganized development. Type I teratomas are likely benign because the improper differentiation is the result of a failure of development rather than the acquisition of an oncogenic mutation.

In contrast to teratomas, YSTs are highly malignant. Old and new insights into PGC development may reveal why this tumor, which is composed of one germ layer, that is, endoderm (or two if one considers embryonic and extraembryonic endoderms separately), takes on a much more traditional tumorigenic phenotype. These tumors histologically resemble many differentiated endodermal structures, possess regions of primitive endoderm histology, and grow rapidly requiring treatment through surgical resection and adjuvant chemotherapy [24, 38]. As mentioned previously, PGC specification is controlled by a concerted effort between SOX17 and BLIMP1, the second of which is responsible for preventing endodermal differentiation [39]. This is required because SOX17 in the absence of BLIMP1 or even in excess of BLIMP1 is responsible for specification of the definitive endoderm, an endodermal structure responsible, through complex differentiation programs, for the development of most adult endodermal structures [40]. Kobayashi et al. recently showed in porcine, monkey, and human systems that even after PGC priming of mesendoderm cells, overexpression of SOX17 or loss of BLIMP1 resulted in differentiation to definitive endoderm [41]. Interestingly, almost 3 decades ago, it was shown in rats that externalization of fetal yolk sac primitive endoderm after fetectomy was capable of formation of malignant YSTs which could be transplanted to syngeneic rats [42]. This provides evidence that primitive endoderm cells have the capacity to grow rapidly and acquire oncogenic lesions which enhance growth when removed from their normal developmental location. Rather than terminally differentiating like their teratoma counterparts, they grow rapidly and act as the source of their own growth regulatory signals, as do many endodermal compartments [43]. This proliferative licensing of primitive endoderm may serve as the explanation for the GWAS SNP associated with HNF1*β*, which is an important transcription factor related to endoderm differentiation and maintenance [44]. Aberrations in HNF1*β* signaling in the context of a PGC may favor primitive endoderm misdifferentiation and subsequent YST formation.

## 6. PGC Migration and Proliferation

After the PGCs have been specified as the independent germ lineage, they must make their way to the gonadal ridge, proliferating en route, where they will receive subsequent differentiation cues for sex-specific development. However, this journey serves as a source of developmental vulnerabilities that may explain the frequent extragonadal localization of pediatric GCTs. After specification of the PGCs in the early mesendoderm ∼2 weeks after conception, they make their way to the wall of the yolk sac endoderm [45]. Subsequently, PGCs migrate along the hindgut and midgut endoderm until near the gonadal ridge (GR) [46]. At this point, they migrate through the dorsal mesentery to the dorsal body wall where they migrate laterally to the GR. This migration generally results in the specific localization of viable PGCs to the GR; however, multiple sections of this tightly controlled migratory pathway present developmental vulnerabilities.

Pediatric germ cell tumors frequently present in extragonadal locations in pediatric patients. These locations are limited to midline structures such as the sacrococcygeal, retroperitoneal, mediastinal, cervical, and intracranial regions. The migratory route coupled with the molecular mechanisms governing PGC migration is likely the causal factor in GCT localization. Though the exact mechanism by which PGC migration is controlled is not fully understood, multiple cytokine mechanisms have been implicated. Interruptions of these pathways have revealed ectopic germ cell localization. The GWAS SNP most strongly correlated to GCT risk resides in the *KITLG* locus. KITLG is the ligand of the KIT tyrosine kinase receptor, which is thought to regulate migration from the midgut to the GR, among other roles [47, 48]. Knockdown of CXCR4 and its ligand CXCL12 results in PGC mismigration and failure to reach the gonadal ridge after PGCs reach the dorsal wall [49]. *β*-integrin and E-cadherin cell surface proteins as well as surface lipids have been implicated in migration along the midgut endoderm [50]. Finally, a study by Runyan et al. revealed that PGCs that fail to exit the midline to enter the gonadal ridge are cleared through a BAX-dependent apoptotic program [51]. Intriguingly, GWAS studies found that an SNP near the *BAK1* locus, which is an important member of the BCL2-BAX-BAK1 antiapoptotic axis, was associated with GCT risk [52]. These observations together reveal the developmental vulnerabilities of PGC migration.

If perturbations of these migratory mechanisms result in failure to appropriately reach the GR, we might expect to find extragonadal GCTs (EGGCTs) in body regions that develop from the tissues along which PGCs migrate. The germ cells must migrate past the dorsal aorta from the dorsal mesentery on the way to the GR. Migration failure at this point would result in PGCs residing along and near the aorta which eventually reside in the mediastinum and the retroperitoneum, two frequent sites of EGGCTs [46]. Anterior-posterior, rather than lateral, mismigration along the body wall may be the mechanism for EGGCTs occurring in the sacrococcygeal and cervical regions. Aberrant migration from the hindgut could explain sacrococcygeal GCT prevalence as this structure is caudal and adjacent to the region which will form the tailbone. Intriguingly, another mechanism based on nerve fibers may explain the distal localization of some EGGCTs. Mollgard et al. found that PGCs migrate along nerve fibers from the midgut along the dorsal mesentery and into the GR. These PGCs associated intimately with the peripheral Schwann cells [53]. These data suggest that cells of the autonomic nervous system (ANS) may be responsible for secreting cytokines which PGCs recognize as migration and proliferative cues. This idea is further corroborated by studies involving the secreted neural signaling molecules PACAP and GDNF, both of which have migratory and proliferative effects on germ cells in vitro [53–55]. These proteins are both secreted by the ANS and could explain the cranial, cervical, and sacrococcygeal localization of ectopic PGCs and EGGCTs as these regions are thoroughly enervated by the ANS. Based on this evidence, further investigation of the link between PGC migration and ANS signaling is warranted.

During the migratory phase of PGC development, their numbers expand rapidly in order to properly colonize the GR. This proliferative step may be a key in the initiation of pediatric GCTs. The primary determinant of this proliferative step is KIT signaling. As PGCs migrate from the midgut to the GR, somatic KIT ligand (KITLG) secretion induces rapid PGC proliferation and suppresses apoptotic pathways used to clear ectopic cells [56]. The signaling axis relating to KIT-controlled proliferation has proven especially interesting due to the fact that two primary interactors have been discovered to have associated SNPs linked to GCT risk. SNP variants near the KITLG have revealed a GCT-associated odds ratio of approximately 2.5 which represents the strongest GCT SNP association to date and one of the highest general cancer associations described [47, 48, 57]. Mahakali et al. showed through a series of mutations to the KITL (murine KITLG homolog) that interruption of KITLG signaling resulted in diminished PGC proliferation [56]. In vitro assays have shown that excess KITLG can induce rapid proliferation of PGCs [58]. The KITLG association with GCTs may thus be due to a variation that causes some increase in KIT signaling, resulting in more robust PGC proliferation. This expansion of the pool would create two independent vulnerabilities to GCT development. Firstly, the rapidly dividing pool of cells is vulnerable to acquisition of mutations that may drive oncogenesis and becomes doubly so when proliferation is increased through excess KITLG secretion. Secondly, KIT signaling activates prosurvival pathways that would allow for expansion of cells that have acquired genetic lesions as well as survival of ectopic PGCs if the range of KITLG secretion was increased by the SNP variants. Further evidence strengthening the relationship of the KIT axis to GCTs is the discovery that an SNP near the gene SPRY4 is associated with GCT risk [47]. SPRY4 is an inhibitor of RTK signaling and has been shown to abrogate KIT signaling [59, 60]. An SNP at this locus resulting in reduced SPRY4 expression could serve a function similar to KITLG increase by failing to reduce KIT signaling. It has also been found that KIT is frequently mutated in intracranial germinomas; however, this finding may be an independent mechanism that helps to drive RAS signaling in Type II GCTs rather than an aberration of normal developmental pathways as would be the case in KITLG-mediated PGC proliferation [61]. This might explain why KITLG is associated with GCT risk for all histologies, while KIT mutations are limited to germinomas. These results suggest that pediatric GCTs are the result of specific aberrations in normal developmental pathways.

## 7. Germline Differentiation

Upon arrival to the nascent gonad, the PGCs must begin the transition from migratory pluripotent cells to either sperm or egg progenitors. This process is controlled through induction of sex-specific gene expression mediated by ligand secretion from the somatic cells of the gonad as well as circulating hormones. The set of developmental processes responsible for sex determination is tightly controlled, and interruption of these differentiation mechanisms represents the final set of vulnerabilities to pediatric GCT development. Work from Looijenga and colleagues has carefully laid out the case that failure of sexual development in the context of disorders of sex development (DSDs) is the proximate cause of the additional GCT risk observed in these patients [62].

As described above, Type II GCTs frequently exhibit a precursor lesion known as germ cell neoplasia in situ (GCNIS) in otherwise normal gonadal tissue adjacent to tumor tissue [9]. These lesions exhibit histological markers, methylation status, and gene expression profiles that are remarkably similar to PGCs, which should normally be absent at the developmental stages at which GCNIS is observed [63]. The molecular mechanisms leading to the development of GCNIS and the transition of GCNIS to invasive cancer, including the possible role of oncogenic mutations, are active areas of investigation. In vitro, PGCs can dedifferentiate to a state resembling a pluripotent embryonic germ cell (EGC), under the control of Akt/PI3 kinase signaling [64]. EGCs exhibit epiblast pluripotency marks such as OCT3/4 and SOX2 and are capable of teratoma formation [65]. Additionally, newly discovered SNP variants of the embryonic pluripotency-related genes ZFP42 and TFCP2L1 have been associated with GCT risk [66, 67]. Taken together, these experimental results emphasize the importance of the OCT3/4-SOX2-SOX17-BLIMP1 network in controlling the fate of developing germ cells. If primitive germ precursors indeed exist in a metastable state between pluripotency and differentiation, perturbations in this network could explain how GCTs can present with such varied histologies.

The unique susceptibility of PGCs to aberrant differentiation may explain the variety of genes implicated in GCT GWAS studies. Rather than traditional SNPs involved in pro-oncogenic or tumor suppressive activities, such as MYC or CDKN1A in colorectal cancer [68, 69], GCT SNPs may cause slight perturbations in PGC development that lead to differentiation failure, mismigration, or aberrant proliferation. This hypothesis is evidenced by multiple mouse models in which the loss of genes responsible for PGC maintenance and eventual sexual differentiation occurs, and testicular teratomas resembling Type I pediatric GCTs result [70, 71]. Two sentinel GWAS loci near the genes DAZL and DMRT1, which are responsible for specification of the germline during sexual development, have also been implicated in teratoma susceptibility [28, 47]. Loss of any of these three genes releases suppression of proliferation and pluripotency in PGCs [72, 73].

## 8. Conclusions

As the only nonsomatic tumor lineage in the body, GCTs exhibit a unique combination of varied histology, wide range of sites of presentation, and apparent lack of traditional oncogenic drivers, suggesting a prominent role for aberrant developmental pathways in the etiology of these cancers. A perspective that focuses on these mechanisms could be key to the development of differentiation-based therapies using either exogenous signaling ligands or small molecule activators or inhibitors of the relevant pathways, which might one day supplement or substitute for conventional cytotoxic therapies. The challenges of long-term adverse effects that arise in the treatment of malignant tumors in young children and adolescents create a powerful incentive for pursuing such approaches.
